# Collagen Assembly at the Cell Surface: Dogmas Revisited

**DOI:** 10.3390/cells10030662

**Published:** 2021-03-16

**Authors:** Moses Musiime, Joan Chang, Uwe Hansen, Karl E. Kadler, Cédric Zeltz, Donald Gullberg

**Affiliations:** 1 Department of Biomedicine and Centre for Cancer Biomarkers, University of Bergen, Jonas Lies vei 91, N-5009 Bergen, Norway; musiime.moses@uib.no (M.M.); cedric.zeltz@wanadoo.fr (C.Z.); 2 Wellcome Centre for Cell-Matrix Research, Faculty of Biology, Medicine & Health, University of Manchester, Manchester Academic Health Science Centre, Manchester M13 9PT, UK; joan.chang@manchester.ac.uk (J.C.); karl.kadler@manchester.ac.uk (K.E.K.); 3 Institute for Musculoskeletal Medicine, University Hospital of Münster, 48149 Münster, Germany; uhansen@uni-muenster.de

**Keywords:** collagen fibrillogenesis, fibronectin, integrin, integrin α11, integrin α2, integrin α5

## Abstract

With the increased awareness about the importance of the composition, organization, and stiffness of the extracellular matrix (ECM) for tissue homeostasis, there is a renewed need to understand the details of how cells recognize, assemble and remodel the ECM during dynamic tissue reorganization events. Fibronectin (FN) and fibrillar collagens are major proteins in the ECM of interstitial matrices. Whereas FN is abundant in cell culture studies, it is often only transiently expressed in the acute phase of wound healing and tissue regeneration, by contrast fibrillar collagens form a persistent robust scaffold in healing and regenerating tissues. Historically fibrillar collagens in interstitial matrices were seen merely as structural building blocks. Cell anchorage to the collagen matrix was thought to be indirect and occurring via proteins like FN and cell surface-mediated collagen fibrillogenesis was believed to require a FN matrix. The isolation of four collagen-binding integrins have challenged this dogma, and we now know that cells anchor directly to monomeric forms of fibrillar collagens via the α1β1, α2β1, α10β1 and α11β1 integrins. The binding of these integrins to the mature fibrous collagen matrices is more controversial and depends on availability of integrin-binding sites. With increased awareness about the importance of characterizing the total integrin repertoire on cells, including the integrin collagen receptors, the idea of an absolute dependence on FN for cell-mediated collagen fibrillogenesis needs to be re-evaluated. We will summarize data suggesting that collagen-binding integrins in vitro and in vivo are perfectly well suited for nucleating and supporting collagen fibrillogenesis, independent of FN.

## 1. Introduction

The extracellular matrix (ECM) is a meshwork of macromolecules which is present as the interstitial matrix and basement membranes. The view of the ECM has changed dramatically during the last few decades. The ECM is composed of large insoluble molecules, and prior to the identification of ECM receptors in the 1980s, scientists claiming that these large insoluble proteins had anything to do with the fine-tuning details of cell biology of importance for tissue homeostasis and pathology, ran the risk of being ridiculed [1]. In the pre-integrin era, the ECM was solely regarded as a structural support. When integrins were identified and it became clear that these cell surface receptors could signal, these views changed. We now know that integrins integrate information from the extracellular microenvironment into most intracellular signaling pathways [1,2,3]. The prediction that ECM is at one end of a dynamic reciprocity, with the cell nucleus where ultimately gene expression is changed at the other end, has thus turned out to be correct [4,5,6].

Historically, the integrin ligands have also had a certain hierarchy with fibronectin (FN) being studied extensively due to ease of availability and documented importance during biological events such as angiogenesis, starting early during development [7,8]. The laminin family, where the biological importance is illustrated by rare genetic diseases in skin and muscle as well as well-documented effects of laminins on cell differentiation and gene expression [9,10,11] has also enjoyed a high status as integrin ligands. Interestingly, in these early studies, the most abundant ECM proteins in vertebrates, the fibrillar collagens, were largely neglected as integrin ligands, with the exception of their role in platelet homeostasis [12,13]. This situation is changing fast, with an increased interest in tissue and tumor fibrosis, tissue regeneration and ageing research, conditions where cell-collagen interactions are recognized to be crucial.

Fibrillar collagens and FN are both synthesized by fibroblasts, and they are both present in interstitial matrices, although with different dynamics. Whereas FN is typically transiently present in acute phases of wound healing and tissue remodeling events, collagens are ECM molecules which are resistant to proteolytic cleavage from broad-spectrum proteases such as trypsin and pepsin, and persist in tissues as major structural components and major determinants of tissue stiffness [14]. Careful studies of composite FN and collagen matrices in vitro have demonstrated that FN binds collagen mainly under low tension, and as the matrix matures, two independent networks form, and the collagen matrix becomes the primary tension bearing element in the mature composite ECM [15,16].

Prior to the identification of the collagen-binding integrins, the general view was that cells interact indirectly with collagens via collagen-integrin bridging molecules (COLINBRIs) such as FN [17,18]. We now know that much of the initial confusion around how cells interact with collagen was related to different cell types having different integrin repertoires with different specificities. Most importantly, when studying cell-collagen interactions in relation to cell-FN interactions, both FN and collagen receptors need to be characterized. Fibroblasts in general express both FN receptors and collagen-binding integrins [19,20]. Although binding of triple helical collagens to the Ⅰ domain of collagen-binding integrins has been firmly established even down to the atomic level [21], the degree to which this binding occurs in mature tissues is more controversial. It has been suggested that, in mature tissues this is a matter of availability of the integrin-binding sites in the densely packed collagen fibrils where the surface of fibrils gradually becomes decorated with interacting proteins and thus shielded from integrin interactions [17,22,23]. A study of intact collagen fibrils from cartilage failed to show binding of these mature collagen Ⅱ containing fibrils to collagen-binding integrins [22]. Interactions of collagen-binding integrins to freshly made collagens, where accessibility and crowding are less of a problem, is likely to take place in this situation.

We think it is time for a critical review of the published work and a fresh look at the role of collagen-binding integrins in collagen matrix assembly. In the current review, we will in some detail discuss different modes of matrix assembly and explain why we think that collagen-binding integrins, independent of FN-binding integrins, will be central in future work aiming at understanding fibrogenic niches, tissue regeneration, tissue remodeling and tissue- and tumor fibrosis.

## 2. Components of the Interstitial ECM

### 2.1. The Collagen Family—Fibrillar Collagens

Collagens make up a family of ECM proteins which are the most abundant proteins in vertebrates. The collagen superfamily is composed of 28 types of collagens, which can be further divided into different subfamilies including: fibrillar collagens, network-forming collagens, fibril-associated collagens with interrupted triple helices (FACIT) collagens, and others [23]. The characteristic feature of a collagen is the presence of collagenous triple helix which can assemble into higher-order structures such as fibrils and networks. The triple helix consists of polypeptide chains with a repeating Gly-X-Y motif, in which the X and Y positions are frequently occupied by proline and 4-hydroxyproline. The fibrillar collagen types Ⅰ, Ⅱ and Ⅲ are the most abundant collagens, and together with the minor collagens type Ⅴ, Ⅺ, ⅩⅩⅣ and type ⅩⅩⅦ they form the fibrillar collagen subgroup [23]. The fibril-associated collagens with interrupted triple helices (FACIT) collagens (collagens Ⅸ, Ⅻ, ⅩⅣ, ⅩⅥ, ⅩⅨ, ⅩⅩ, ⅩⅪ and ⅩⅫ) do not form fibrils themselves but they, as the name suggests, associate with fibrillar collagens. Vertebrates have developed an endoskeleton with bone, cartilage and tendons which are all rich in an interstitial matrix where fibrillar collagens are major building blocks. The network forming collagens, including collagen type Ⅳ, provide scaffolds for networks such as basement membranes (BM) [24]. In addition to providing structural support, some collagens can also bind to cell surface receptors such as discoidin-domain receptors (DDRs), glycoprotein Ⅵ, osteoclast-associated receptor (OSCAR), Leukocyte-associated immunoglobulin-like receptor 1 (LAIR-1), G protein-coupled receptor 56 (GPR56; recently shown to play an important role on platelets) [25], and most relevant to this review, the collagen-binding integrins α1β1, α2β1, α10β1 and α11β1 [19]. Syndecans are also able to act as co-receptors for ECM proteins such as FN and collagens [26,27]. Signaling by these different receptors can influence transcription and cell behavior in multiple ways [19]. The different cell surface receptors are often, but not always, able to form direct link between the ECM and the cell cytoskeleton. Some of the proteins that are listed as collagen receptors appear to mainly function in signaling rather than acting in mechanical linkages. The integrin-collagen interaction constitutes a robust mechanical link which, in addition to being able to take part in cell signaling, is also involved in cell adhesion, cell migration and remodeling of the collagen matrix [19]. These functions are essential for tissue homeostasis and are receiving increasing attention as we learn more about the detail of tissue and tumor fibrosis, tissue regeneration and tissue ageing. The role of collagen-binding integrins in the actual assembly of collagen fibrils is more controversial [17,28], and will be discussed below.

### 2.2. Fibronectin

Fibronectin (FN) is a large modular extracellular matrix protein, composed of two polypeptide chains (each with a Mw of approximately 220 kD) which are linked by disulfide bonds and composed of type Ⅰ, type II and type Ⅲ repeats [29,30]. FN RNA is alternatively spliced at three conserved regions EⅢA (EDA), EⅡB (EDB) and Ⅴ (CS-1) to generate 20 different protein variants in humans [31]. These different variants are present in two forms: soluble plasma FN (pFN) and cellular FN (cFN), and bind to integrins, heparin, collagens, and fibrin via distinct domains [30]. FN contains the integrin binding RGD site in the 10th FN type Ⅲ repeat, but a so-called synergy site at 9th type III repeat is needed for optimal binding of the α5β1 integrin and efficient FN fibrillogenesis [32]. α5β1 is the major integrin involved in FN fibrillogenesis, but other FN-binding integrin receptors like αvβ3, α4β1 can also take part [33,34]. More recent data suggest that, in the context of basement membranes the integrins α2β1 and α3β1 can play a supporting role in α5β1-mediated FN fibrillogenesis [35]. The regions in FN involved in binding gelatin and collagen are found in FNI_6_, FNII_1–2_ and FNI_7–9_, present in the 70 kD N-terminus of FN [36,37,38]. There is no evidence that either EDA or EDB regions directly bind to collagens, but they will be discussed in some detail since FNs containing these domains are the FN variants which interact with fibrillar collagens in tissue and tumor fibrosis. The EDA and EDB domains display 29% sequence identity, but are each highly conserved among vertebrates [31]. Whereas a number of receptors have been described for EDA, the cellular receptor(s) for the EDB domain remains largely unknown. Therefore, most of the focus related to these splice forms has been on the EDA isoform. The EDA and EDB isoforms are both highly expressed during embryonic development, especially in developing blood vessels [39], but are almost absent in the adult organism where vascularization and tissue reorganization is quiescent. During wound healing [40], fibrosis and in solid tumors [41], the EDA/EDB embryonic splice variants are re-expressed [42], leading to the term “oncofetal” splice variants sometimes being used. Some studies suggest that these embryonic splice forms in tumors are mainly expressed in neo-vasculature [43], whereas other studies demonstrated their presence in the fibrotic stroma associated with myofibroblasts [44,45]. Early studies suggested that the presence of EDA in intact FN indirectly influenced the exposure of the RGD sequence in the 10th FN type Ⅲ repeat leading to higher binding affinity for integrin α5β1 to EDA FN [46], similar to the role of the synergy site. In separate studies it has been demonstrated that integrin α9β1 and α4β1 can bind directly to a cryptic loop region in an EDA containing fragment, but not to the intact EDA FN [47]. α4β7 integrin on lung fibroblasts has also been shown to bind directly to EDA FN [48]. Similarly, Toll-like receptor 4 (TLR4) has been reported to be activated upon binding to the isolated EDA fragment, but not upon binding to the intact EDA FN. Binding of these receptors to the cryptic site thus requires proteolytic cleavage of FN. In summary, EDA FN is highly expressed in granulation tissue, in fibrotic lesions and in the tumor stroma. FN variants hat include EDA and EDB regions—commonly termed cellular FNs—are prominent in these pathological settings and these also interact with fibrillar collagens in these matrices. Critical analysis in genetic models demonstrated a moderate effect of EDA FN in wound healing, but with new methods and more careful analyses in new experimental genetic models important contributions to fibrosis and tumorigenesis are increasingly being recognized [49,50]. The suggested crucial role of FN in collagen fibrillogenesis will be discussed later.

### 2.3. Tenascins and Other ECM Proteins in the Interstitial Matrix Influencing Collagen Organization

Matricellular proteins are secreted macromolecules that do not play a primary role in matrix structure, but that are able to modulate cell interactions and functions [51,52]. The matricellular protein family includes thrombospondins, tenascins, SPARC (secreted protein acidic and rich in cysteine), periostin, osteopontin and the CCN (CYR61, CTGF, NOV) family of proteins. In this review, we focus on the role of tenascins and periostin, but also briefly discuss proteoglycans.

#### 2.3.1. Tenascins

The tenascin family is composed of four members in vertebrates: tenascin-C (TN-C), tenascin-R (TN-R), tenascin-X (TN-X) and tenascin-W (TN-W), which are expressed in different tissues with a common role in modulation of cell adhesion and spreading [53]. Of these, the TN-C isoform has been studied the most. TN-C is absent or expressed at low levels in adult tissues, in contrast to the strong expression observed in fibrosis and cancer. Just as the Toll-like receptor 4 (TLR4) has emerged as a receptor mediating pro-fibrotic signals for EDA FN, TLR4 has also emerged as a receptor binding to different tenascin isoforms. A detailed study focusing on different motifs in TN-C has identified a structure in fibrogen-like globe domain (FBG) of TN-C that is predicted to be active in TLR4 binding also in TN-R- and TN-W, but notably not in TN-X [54]. A number of studies suggest that TN-C effects are mediated by both integrins and TLR4 receptors, often creating a complex interaction network involving paracrine signaling [55]. Experiments using cell cultures and experimental fibrosis in mice have demonstrated a role for TLR4 in TN-C-dependent skin and lung fibrosis [56]. With regard to the cellular mechanisms whereby TN-C mediate its effects, a study using human mammary fibroblasts as a model of breast cancer-associated fibroblasts (CAFs) suggests that TN-C treatment increased collagen gel contraction and increased synthesis of TN-C and integrin αvβ1, in turn leading to increased TGF-β activation [57]. This is suggested to be a mechanism promoting increased matrix stiffness. It will be interesting to pursue how TN-C mediates this effect on collagen gel contraction. Since this process ultimately depends on a stable link between cells and the collagen matrix, it is possible that the cell-TN-C interaction creates a stimulatory autocrine signal strengthening the link between collagen-binding integrins and the collagen matrix.

Tenascin-X is the tenascin isoform most relevant for collagen assembly. It is expressed in several tissues, with high expression in skin and skeletal muscle [58]. Deficiency or mutation in TN-X gene leads to a form of Ehlers–Danlos syndrome (EDS), characterized by skin and joint hyperextensibility [59]. At the ultrastructure level absence of TN-X leads to a collagen matrix with larger fibril diameter and larger inter-fibrillar spacing [60]. It has been suggested that TN-X regulates collagen fibril spacing both by direct binding via C-terminal end of TN-X molecule to collagens Ⅰ, Ⅲ and Ⅴ [61], and indirect binding to collagen fibrils, mediated via decorin [62]. Whereas other tenascin isoforms contains active RGD sites, TN-X lacks RGD sequences that are unmasked and available for cells to bind in the intact TN-X molecule [63]. Alcaraz et al. have suggested a role for TN-X in breast cancer progression through TGF-β activation and epithelial-to-mesenchymal transition (EMT), which is suggested to be dependent on α11β1 integrin [64]. It will be interesting to determine if the binding of α11β1 to TN-X is direct, and if so, which part of integrin α11β11 binds to TN-X, since TN-X lacks a collagen triple helix domain that typically includes the GFOGER motif, the peptide within collagens that binds to α11β1 I-domain.

#### 2.3.2. Periostin

Periostin is a matricellular protein, which is highly expressed in mesenchymal tissues during development [65]. Periostin is a homodimeric matricellular protein belonging to fasciclin family. Like TN-C, periostin is induced in the tumor stroma. Detailed studies has revealed complex interactions with αv integrins (αvβ1, αvβ3, αvβ5) [66]. Genetic deletion of periostin leads to tooth defects and a periodontal-like disease, which result in dwarfism [65]. Wound healing studies suggest a promoting effect of periostin in dermal myofibroblast differentiation and collagen gel contraction [67]. A pro-fibrogenic role for periostin in cardiac and skeletal muscle fibrosis has also been reported [68,69]. Periostin interacts with fibrillar collagens, although the exact domain has not been mapped [66]. In the absence of periostin the collagen fibrillar diameter increases [70,71].

#### 2.3.3. Stromal Proteoglycans

Proteoglycans (PGs), abundant at cell surfaces and in the ECM, belong to a group of glycoproteins in which the core protein is substituted with one or more polysaccharide chains (called glycosaminoglycans; GAGs). Heparan sulfate (HS) PGs execute their function by binding to a variety of molecules including members of several growth factor families, chemokines, morphogens, serine protease inhibitors, and extracellular matrix proteins. Syndecans are transmembrane HSPGs with four members in vertebrates, syndecans 1–4 [72,73]. When present at the cell surface they are formally not part of the interstitial ECM, but can affect ECM assembly in both unshed and shed forms. They are involved in diverse biological processes, such as regulating cell adhesion, cell migration and differentiation as well as participating in the organization of ECM and the cytoskeleton [74]. Syndecans can serve as co-receptors in various signaling pathways on the cell surface and also provide a link between the ECM and the cytoskeleton by directly interacting with the cytoskeleton or via other molecules [72]. Syndecan-4 is ubiquitously expressed at low levels. Although integrins are the canonical mechanotransducing cell surface receptors, syndecans have also been regarded to take part in mechanosensing through their role as co-receptors for collagen- and FN-binding integrins [26,27]. It is in this role as co-receptors that syndecans have been suggested to have a role in matrix assembly. Recent studies suggest that syndecan-4, rather than only being present with integrins in the same adhesion sites, can also generate signals in response to tension (at subcellular sites separate from integrin adhesions) that activates the kindlin-2/β1 integrin/RhoA axis in a PI3K-dependent manner [75]. These new results have been obtained in an advanced experimental in vitro system using FN- and collagen-coated magnetic beads. It will be interesting to see if the proposed model of syndecan-4 mechanosignaling activating integrins is also valid under more in vivo-like 3D conditions. One interesting feature of syndecans is the shedding of the extracellular domain that enables syndecans to act as soluble factors [76]. The shedding occurs next to the plasma membrane and is processed by different MMPs: MMP-7 is involved in syndecan-1 and -2 shedding, MMP-2 and -9 can cleave syndecan-1, -2 and -4, whereas MMP-14 can cleave syndecan-1 and -4 [77,78,79]. Syndecan-1 has been suggested to take part in FN fibrillogenesis by activating integrins [80], whereas a similar role for direct collagen-binding integrin-mediated collagen fibrillogenesis has not been shown although syndecan-1 has been shown to cooperate with α2β1 integrin in collagen binding [81]. Intriguingly, in dermal fibroblasts the presence of syndecan-1 has the opposite effect on FN fibrillogenesis, suggesting that shedding events may influence some autocrine loop needed for the syndecan-1-integrin axis of FN assembly. Finally, when fibronectin-mediated collagen assembly was studied using the embryonic *Fn*^−/−^ cells and FN with a mutated RGD site was added to these cells, the mutated FN still assembled onto cells (in an α5β1 integrin-independent manner) [82]. This assembly could be blocked by heparin, strongly suggesting involvement of syndecan HS chains.

Small Leucine-Rich Proteoglycans (SLRPs) are ECM proteins rich in leucine-rich repeats, conferring a “banana” shape structure with a concave face involved in protein-protein interactions. For decorin, leucin rich repeats 4–6 are involved in collagen-binding [83]. Most SLRPs binds to fibrillar collagen and regulate collagen fibrillogenesis and matrix assembly [84]. A recent study illustrates that the type of assay is very important for demonstrating effects of SLRPs on collagen organization and reorganization [85]. Oldberg et al. have shown that in experimental carcinomas, fibromodulin promotes the formation of a dense collagen matrix through the regulation of fibril diameter, leading to an increased interstitial fluid pressure (IFP), with possible adverse consequences for delivery of chemotherapeutics [86]. It is interesting to remember that other SLRPs also modulate collagen fibrillogenesis and could thus be involved in IFP regulation in different types of cancers, despite their anti-tumorigenic properties [87]. SLRPs also function to sequester TGF-β [88], a growth factor already involved in EMT and fibroblast activation contributing to tissue and tumor fibrosis. In this way SLRPS can indirectly influence matrix assembly by regulating levels of both ECM molecules and ECM receptors.

## 3. Mechanisms of ECM Assembly

With the increased awareness about the importance of the status of the ECM (composition, organization and stiffness), there is also a mounting interest in finding good assay systems to evaluate the influence of various parameters on matrix assembly and understand the molecular mechanisms involved. For in vitro systems 2-D versus 3-D ECM, soft versus stiff ECM, serum-containing versus serum-free cell culture medium, inclusion of ascorbic acid or no addition of ascorbic acid, spheroid culture with one cell type (homotypic model) or multiple cell types (heterotypic models), are all important parameters. The issue with serum, with regard to cell-ECM interactions; deals with the presence of FN at relatively high concentrations in this cell culture supplement [30], but also the presence of other ECM proteins including vitronectin [89] and collagen Ⅵ [90]. When comparing data evaluating the role of matrix assembly, and in particular collagen assembly, all these parameters have to be kept in mind, together with the consideration of the varied integrin repertoire of the cells studied during the course of the experiments. In a recent detailed study, the effect of different proteoglycans on collagen organization was evaluated with a broad spectrum of assays [85]. It is also important to remember that the dynamics of the experimental system will also have effects on ECM assembly. Although the studies of cell-FN interactions have greatly advanced our understanding of integrins, in the context of collagen-binding integrins, the presence of FN creates a problem when trying to study direct cell-collagen interactions at the cell surface. When studying cell-collagen interactions, the presence of FN is thus a “disturbance” in most in vitro cell culture conditions, which can easily “overshadow” the cell-collagen interaction. However, with new technologies allowing efficient inactivation of genes, a new window for studying FN-free cell-collagen interactions at the cell surface has been opened.

### 3.1. Fibronectin Fibrillogenesis—Integrin Initiated Assembly, a Central Mechanism for Matrix Assembly in Cell Culture

Fibronectin is one of the most studied ECM proteins both with regards to cellular activities such as cell adhesion, cell migration but also with regards to ECM assembly. Binding of FN to the classical FN receptor α5β1 integrin via the RGD and synergy site in FN Ⅲ9-10 leads to a conformational change in FN exposing sites needed for fibril formation [91]. RhoA and the actin/myosin Ⅱ cytoskeleton is also needed to exert a pulling force on α5β1 integrins to move them and FN from focal adhesions into fibrillar adhesions [92]. The phosphorylation of proximal NPXY motif in β1 integrin lowers the affinity to talin which is suggested to result in replacement of tensin, the so called talin-tensin switch [93,94]. These are the core events of FN fibrillogenesis. During the assembly process integrins are thus pulled from focal adhesion into fibrillar adhesions. The “fibrillar adhesion” is a specialized type of cell-ECM interaction renamed fibrillar adhesions from “ECM contacts” more than 20 years ago [95]. The current definition of fibrillar adhesion is that it is a specialized form of cell-ECM adhesions that translocate centripetally from peripheral focal adhesion to facilitate the formation of FN fibrils [92]. As pointed out in a careful study of matrix assembly in young nascent matrices vs. mature established matrices, the role of individual ECM components and their cognate receptors in these matrices varies [96]. In nascent matrices FN can, under some conditions, act as a scaffold for the assembly of a complex matrix; thus disrupting FN fibrillogenesis will disrupt assembly of new matrix at cell surface. In mature matrices, a number of matrix contacts are already assembled, and the distinction between incorporation into an ECM in a cell surface-dependent manner or cell surface-independent manner becomes blurred [96]. A number of additional aspects of FN fibrillogenesis have been examined in detail. Fibrillar adhesions and FN assembly have been demonstrated to be regulated by the AMP-activated kinase (AMPK) which inhibits FN fibrillogenesis [97], the adapter protein ILK [98], the cytoskeletal proteins tensin-1 and -3 [92], the cytoskeletal protein Hic-5 [99] and the small GTPases Cdc42 (promotes) and RhoJ (represses) [100,101] to regulate their composition or formation. It is interesting to note that although deletion of individual tensins or ILK change the composition of the fibrillar adhesions it does not affect α5β1 integrin localization or FN fibrillogenesis [99] (Table 1).

**Table 1:** Proteins Involved in Fibronectin Fibrillogenesis In Vitro.

Protein	Type	Role in Fibronectin (FN) Fibrillogenesis In Vitro	Reference
α5β1 integrin	Membrane receptor	Primary FN assembly receptor.	[102]
α4β1 integrin	Membrane receptor	May contribute to FN assembly.	[33]
αvβ3 integrin	Membrane receptor	May contribute to FN assembly.	[8,34]
Syndecan-1 (SDC1), syndecan-4 (SDC4)	Membrane receptors	SDC1 implicated in FN fibrillogenesis. SDC4 Co-receptor for integrins, but also signaling cross-talk. Heparin can inhibit FN fibrillogenesis.	[77,82,84]
AMP-activated protein kinase (AMPK)	Kinase	Repress fibrillogenesis by negatively regulating tensin-dependent integrin activity.	[97]
Integrin linked kinase (ILK)	Adapter protein, pseudokinase	Promote fibrillogenesis by stimulating focal adhesion maturation and fibrillar adhesion formation.	[98]
RhoA	Small GTPase	Allow formation of α5β1 containing fibrillar adhesions.	[103]
RhoJ	Small GTPase	Repress fibrillogenesis by diverting α5β1 integrin into degradative fate.	[100]
Cdc42	Small GTPase	Stimulates FN fibrillogenesis in endothelial cells in vitro.	[101]
Tensins	Cytoskeletal protein	Bind to phosphorylated proximal NPXY motif in integrin β1 chain with higher affinity than talin.	[92]
Hic-5	Scaffolding protein	Promote fibrillogenesis by stabilizing tensin-β1 integrin interaction.	[99]

### 3.2. Laminin Assembly—Uncontroversial Roles of Sulfatides, Integrins and Dystroglycan

Unlike FN where the plasma form of FN is a globular protein unable to initiate assembly into fibrils without the help of cells, the sites needed for formation of higher order structures are not hidden in the major fibrillar collagens or in laminins [17,19,30]. These ECM molecules can thus self-assemble in entropy-driven process in vitro in the absence of cells. However, for both basement membrane laminins and fibrillar collagens matrices the assistance of cells is needed in order for the ECM to be deposited in an organized manner corresponding to the local tissue needs (Table 2).

**Table 2:** Integrins Implicated in ECM Matrix Assembly.

Ligand	Integrin	Cells	References
**Fibronectin**			
(matrix assembly in vitro)	α5β1	Fibroblasts, stromal cells	[102]
	αvβ3	Mouse embryonic cells	[8,34]
	α4β1	RAMOS B-Cells, α4 integrin-transfected CHO cells	[33]
**Laminins**			
(binding/matrix assembly in vitro)	β1	Mouse embryonic stem cells	[104]
	α3β1	Endothelial cells	[105]
	α6β1	Epithelial cells, endothelial cells	[106]
	α7β1	Muscle cells	[107,108]
**Collagen Ⅰ/Ⅲ**			
(matrix assembly in vitro)	α2β1	Vascular smooth muscle cells, mouse embryonic cells transfected with collagen-binding integrins	[109,110]
	α11β1	Mouse embryonic cells transfected with collagen-binding integrins	[110]
**Collagen Ⅴ**			
(binding)	α2β1	Human epithelial cells	[111]
	α11β1	Mouse embryonic fibroblasts	[112]

In vitro experiments have shown that laminin networks can form in a cell-dependent manner using laminin receptors including the laminin-binding integrins α3β1 and α6β1 [104,113]. The role of cell surface sulfatides to concentrate laminins and enable dystroglycan and integrins to nucleate and assist in laminin network assembly is now well accepted [104,113]. As already mentioned, the laminin-binding integrins α3β1 and α2β1 (α2β1 integrin is a default collagen-binding integrin [114], but can also bind laminins [115] (although binding sites for laminins in the integrin α2 chain has not been characterized)) have also been demonstrated to be indirectly involved, by perform an anchoring function during the assembly of a FN matrix [35]. The unorthodox role of laminin binding-integrins to initiate FN-assembly has been suggested to explain the concentration of FN close to basement membranes that can be observed during embryonic development and which is present in several adult organs [35].

### 3.3. Cell Surface—Mediated Collagen Fibril Assembly—Direct or Indirect Integrin Links

Just as laminin assembly has been shown to occur at cell surfaces it has been recognized that cell surfaces assist in collagen assembly, and this view is also no longer controversial [28,110]. The advantage of studying collagen fibrillogenesis is that the D-periodic fibrils are readily recognized by electron microscopy. Early work by Trelstad [116] then later by Birk [117] and Canty [118] showed that the tips of D-periodic collagen fibrils are contained within plasma membrane invaginations (known as fibripositors), especially in embryonic tissues that are actively assembling collagen fibrils. Although volume electron microscopy has shown that assembly of collagen fibrils at the plasma membrane establishes the template for fibrous tissue expansion [119] this approach has not been able to identify other macromolecules that might participate in the assembly process. Thus, the molecular mechanism of cell-surface assisted collagen fibrillogenesis, both in vitro and in vivo, remains unclear. Is FN crucial, or is the view of FN being central biased by in vitro culture conditions? The central questions about collagen fibrillogenesis at this stage are thus:

1. To what extent do cells take advantage of the cell surface assembled FN (α5β1-mediated) for collagen I fibrillogenesis? Is the contribution of FN to collagen fibrillogenesis more of an in vitro “phenomenon” than a general in vivo mechanism?

2. How common is the direct integrin-mediated assembly of collagen Ⅰ fibrils which takes place independent of FN? (α?β1-mediated)?

3. What exactly is the role of collagen Ⅴ with regard to integrin interactions during collagen fibrillogenesis?

These questions were already posed by Kadler et al. in 2008 [28]. Although we still do not have the answers to these questions more than a decade later, a few promising developments have taken place since 2008. Namely, a new role for FN in facilitating procollagen cleavage at the cell surface has been established [120]. In addition, some of the collagen-binding integrins with a role in tissue and tumor fibrosis as well as wound healing have become more interesting as potential main players in collagen matrix assembly [121,122,123,124]. Finally, new technology has offered new tools to experimentally address some of the central issues, i.e., enabling studies of collagen fibrillogenesis in the absence of a FN matrix [125]. We thus anticipate that further studies of cells possessing collagen receptors but lacking the major FN receptor integrin α5β1 will generate valuable results that will increase our understanding of the major players during collagen fibrillogenesis in coming years [125].

#### 3.3.1. Fibronectin as the Main Player in Collagen Fibrillogenesis: Indirect Mode of Assembly

**Figure 1:**
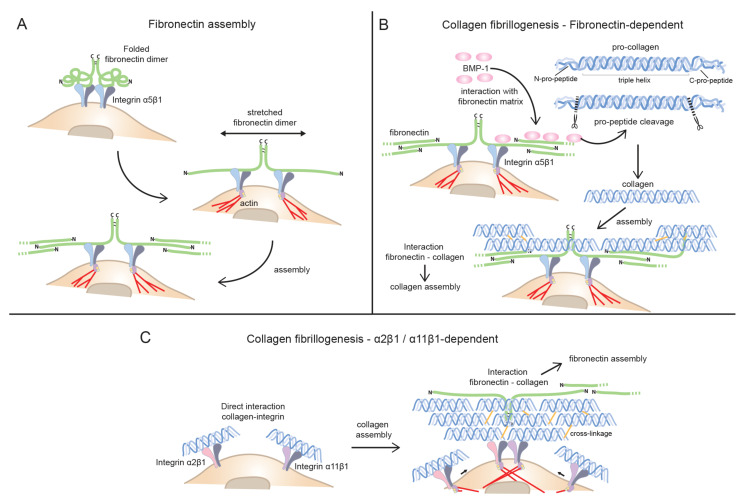
Collagen and fibronectin fibrillogenesis. (**A**) Fibronectin fibrillogenesis. Binding of fibronectin (FN) to the FN receptor α5β1 integrin leads to a conformational change in FN exposing sites needed for fibril formation. The actin cytoskeleton exerts a pulling force on α5β1 integrins to unfold FN. (**B**) Indirect mode of collagen assembly (fibronectin-dependent). Fibrillar collagens are secreted as pro-collagen. FN facilitates collagen assembly by concentrating BMP-1, which is involved in collagen pro-peptide cleavage. Collagen could then interact with FN matrix that serves as nucleator of collagen assembly. (**C**) Direct mode of collagen assembly (collagen-binding, integrin-dependent). The collagen-binding integrins α2β1 and α11β1 directly interact with collagen. They have been suggested to assist in collagen assembly. The FN and collagen matrix assembly are closely linked. These integrins might have a central role in the conditions where FN assembly is dependent on collagen fibrillogenesis.

As already mentioned, FN assembly is critically dependent on integrin receptors like α5β1 to initiate the process of FN fibrillogenesis. FN bound to integrins undergo a conformation change enabling FN self-assembly, as described [91] (Figure 1A). In mature ECM matrices the further addition of FN does not need α5β1 integrin or other integrins to be added into network [96].

Historically, in vitro studies have demonstrated a close association between FN and collagen assembly [126,127,128]. Studies of embryonic Mov13 cells, lacking collagen Ⅰ containing fibrils, surprisingly also revealed a reduced FN matrix in absence of the collagen matrix [129]. Transfection with collagen α1(I) cDNA rescued collagen and FN matrix assembly but attempts to rescue with cDNA encoding a mutated collagenase cleave site (aa 774 and aa 777 of α1(Ⅰ) surrounding actual cleavage site at aa 775–776, were mutated) failed to rescue FN assembly, confirming the collagenase cleave site as the key interaction site in collagen Ⅰ needed for collagen-mediated FN fibrillogenesis. When the corresponding experiments were performed in FN, i.e., deleting collagen binding site FN ΔI6-9, FN failed to assemble collagen I in cells lacking collagen receptors [130].

Similarly, studies in smooth muscle cells showed that blocking α5β1 integrin with function-blocking antibodies, blocked both FN and collagen fibrillogenesis, whereas blockage of α2β1 in the same cells had a more a partial effect [109]. In another set of experiments, studies have been performed using *Fn^−/−^* mouse cells. These studies include cells derived from *Fn*^−/−^ embryonic stem cells [131] with unknown repertoire of collagen receptors, and MEFs isolated from *Fn*^−/−^ mice [132]. Partial analysis of collagen-binding integrins on *Fn*^−/−^ MEFs revealed lack of collagen-binding integrin α2 chain, which is normally found in MEFs [132,133]. In some studies clone 4D cells have been used. These mouse embryonic cells have been demonstrated to lack the collagen receptors α2β1 and α11β1 [110]. The lack of collagen receptors on the *Fn*^−/−^ MEF cells might be related to early embryonic lethality of *Fn*^−/−^ mice necessitating the isolation of MEFs at E8–E8.5 instead of the standard stage of E13.5–E14.5 [134]. The nature of these embryonic cells is thus somewhat unclear since they lack major integrin collagen receptors usually present on fibroblasts [110,133]. These embryonic *Fn*^−/−^ mesenchymal cells have however been used in numerous studies to claim that collagen fibrillogenesis and collagen gel contraction are dependent on a FN matrix [130,132,135]. We do not know the exact reason why these cells show such a dependence on FN for collagen fibrillogenesis and collagen gel contraction, but it is possible that this might simply be because these cells lack collagen receptors. Claiming that FN is needed for collagen fibrillogenesis, might only apply to cells lacking biologically relevant levels of collagen-binding integrins. Experiments with clone 4D cells mentioned above support a role of α2β1 and α11β1 in collagen fibrillogenesis, provided these integrins are expressed [110]. Whereas genetically engineered cells thus might lack collagen receptors, fibroblasts in their physiological environment in vivo do have collagen receptors. When extrapolating results and drawing conclusion about the relative importance of FN and collagen-binding integrins for collagen fibrillogenesis, it is thus crucial to keep track of the collagen receptor repertoire of the cells studied.

A second major bias for most in vitro experiments studying matrix assembly relates to the high concentrations of serum-derived FN, which is not the situation in most tissues with intact blood vessels. Long term culture in 10% bovine serum might furthermore increase levels of FN receptors on the cells studied. Just as the relative importance of FN for collagen fibrillogenesis in vitro is unclear (highly dependent on the integrin repertoire of cells studied), the relative importance of FN for collagen assembly in vivo is unclear. Few attempts to address this experimentally has been pursued, but in a careful study using a mouse model of liver fibrosis FN was conditionally deleted in the liver [136]. When fibrosis in this model was induced with CCl_4_, fibrillar collagen matrix assembly took place perfectly normally in the absence of FN [136]. Although integrin α11β1 has been implicated in fibrogenic functions in cultured stellate cells and could thus potentially play a role in collagen fibrillogenesis in the liver, it is unclear if this integrin has similar functions in liver in vivo [137].

Fibrillar collagens are subject to a complex biosynthesis involving secretion of a triple helical molecule (procollagen) with globular (non-triple helical) domains still present [23]. In order for collagen to form fibrils, these domains have to be removed. Whereas cleavage of C-terminal domains is central, retention of amino-terminal domains has been described in some fibrillar collagens. FN has recently been described to aid in BMP-1 mediated cleavage of carboxy terminus of collagen Ⅰ [120]. Once freed of pro-peptides, collagens are assembled. In agreement with this, the FN and BMP-1 -mediated cleavage of pro-peptide facilitated collagen assembly (Figure 1B). This might be an important function of FN on the cell surface that will affect both direct (collagen binding integrin-mediated) and indirect (FN-mediated) collagen fibrillogenesis.

In summary, integrin α5β1-initiated FN fibrillogenesis at the cell surface may act by facilitating collagen assembly by concentrating BMP-1 needed for collagen pro-peptide cleavage [120] and binding tropocollagen-subsequent to FN fibrillogenesis-thus nucleating collagen assembly [17,19,30].

#### 3.3.2. Collagen-binding Integrins in Collagen Fibrillogenesis: Direct Mode of Assembly

Which collagen receptors can cells use to assemble collagens without the assistance of FN? Integrin α1β1 displays a higher affinity for basement membrane collagen Ⅳ than collagen Ⅰ [138], and is unlikely to take a direct part in assembly of a fibrillar collagen Ⅰ matrix on stromal cells. A role of α1β1 in controlling collagen Ⅰ synthesis has been described and it might thus influence levels of assembled collagen Ⅰ [139]. The three remaining obvious candidates are α2β1 and α10β1 and α11β1, which all prefer fibrillar collagens [19]. It would seem that the most rational and economical way for cells to assemble collagen would be via binding collagen receptors directly. Although fibroblasts in general express both α2β1 and α11β1, subsets of fibroblasts in mice also have been reported to express α10β1 [140]. No data exist to suggest a role of α10β1 in collagen fibrillogenesis but it is tempting to speculate that chondrocyte α10β1 could play a role in assembling a collagen Ⅱ-containing matrix in cartilage and that it could assist in assembling a collagen Ⅰ-containing matrix in junctional fibroblasts. Dogs with a mutation in *ITGA10* display chondrodysplastic features in extremities [141]. It would be interesting to analyze collagen organization in affected tissues in these dogs, although disease progression most likely cause secondary changes, unrelated to possible collagen assembly defects due to absence of functional integrin α10β1.

In addition to integrins vertebrates possess multiple collagen-binding receptors [19]. Discoidin domain receptors (DDRs) are tyrosine kinases which are activated by collagens [142]. DDR1 is expressed in epithelial cells in contact with collagen Ⅳ and is thus most likely not a major player in the assembly of fibrillar collagens as described in this review. DDR2 is expressed in stromal cells but has not been reported to form stable cytoskeletal connections as described for DDR1 [143] and is thus most likely not directly involved in the collagen assembly described herein. A study of DDR2 in cancer-associated fibroblasts instead reveal a role in activation of β1 integrins, in turn leading to effective matrix organization [144]. These data suggest that DDR2 might be a major regulator of integrin-mediated collagen assembly and remodeling on stromal cells in tissue- and tumor fibrosis.

The precise mechanism of how a collagen-binding integrin might participate in fibril assembly has not been considered. For example, if an integrin binds a monomer of triple helical collagen, how does this monomer participate in fibril formation? Presumably the relative strength of association of the collagen molecule with the integrin would be an important factor in determining the thermodynamics and kinetics of fibril assembly. Steric displacement of the integrin by the ‘incoming’ collagen molecule might be expected to release the nascent fibril from the cell unless a hand-over-hand mechanism was in place to ‘keep hold’ of the fibril as a new collagen molecule is added to the growing fibril tip. The participation of FACITs, minor collagens like collagen Ⅴ and Ⅺ, collagen-binding proteoglycans, or enzymes that cross-link collagen molecules (e.g., lysyl oxidases) might also facilitate collagen fibril assembly at the cell surface. Finding answers to these and related questions is likely to be one of the most challenging problems of the next decade of cell-matrix research and may require new approaches involving cryo-electron microscopy and super resolution microscopy.

It is worth remembering that for FN, not any FN-binding integrin will take part in FN fibrillogenesis. Why a specific integrin is especially well-suited FN assembly reflects integrin alpha chain specific functions. It is not known if a similar preference exists for collagen-binding integrins, i.e., that a specific collagen-binding integrin heterodimer is better suited than other collagen-binding integrins for collagen fibrillogenesis.

In addition to assisting in collagen assembly the collagen receptors can also be used to remodel a complex collagen matrix. If cells lack collagen receptors, an indirect linkage to FN would mediate collagen fibrillogenesis and collagen re-arrangement. In tissues with abundance of collagen, we predict that collagen-binding integrins might be the preferred choice for collagen assembly and remodeling. As already mentioned, studies of smooth muscle cells [109], and embryonic *Fn*^−/−^ cells [110] have suggested a role of α2β1 and α11β1 in assisting in collagen assembly in vitro (Figure 1C). Studies of fibroblasts from EDS patients with mutations in collagens III and collagen V (resulting in matrix with reduced levels of these collagens) also suggest a role of α2β1 integrin in collagen matrix assembly in vitro [145]. The potential role of α11β1 integrin in collagen assembly has not been evaluated in detail yet. Interestingly, studies of EDS fibroblasts also hint at a complex mechanism involving crosstalk of integrins, suggesting a word of caution when trying to ascribe functions to specific integrins in this system. What we know so far from studies in EDS fibroblasts is that lack of normal synthesis of collagens can lead to changes in synthesis of cognate collagen-binding integrins but also result in a switch of synthesis in FN-binding integrins, like α5β1 switching to αvβ3 [145].

#### 3.3.3. Role of Collagen Ⅴ/Ⅺ in Collagen Ⅰ Fibrillogenesis?

**Figure 2:**
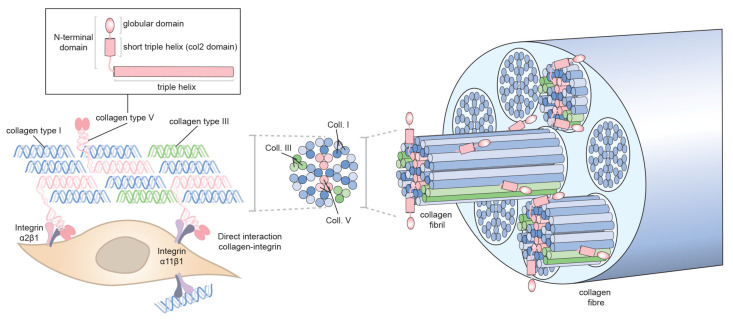
Collagen Ⅴ in collagen I-containing fibrils. Collagen Ⅴ is a minor fibrillar collagen composed of a triple helical part and a N-terminal domain, which is only partially processed, formed by a globular domain and the col2 domain, which is a short triple helix. Collagen Ⅴ (pink) plays a role in nucleating assembly of collagen Ⅰ (blue) matrix, which may also contain collagen Ⅲ (green). Fibrillar collagens are assembled into microfibrils that aggregate to form fibrils and then fibers. Collagen Ⅴ triple helix is hidden in the interior of the fibril with the N-terminal domain protruding at the fibril surface. Collagen-binding integrins at the cell surface may potentially interact with the short triple helix present in the N-terminal domain of the collagen Ⅴ during the formation of fibrils.

For fibrillar collagens, the complexity does not end with the procollagen propeptides being cleaved off extracellularly, enabling fibrillogenesis [23]. The collagen fibrils are heterotypic ‘alloys’ [146] comprising more than one type of fibril-forming collagen [147]. For example, the fibrils in cartilage contain collagen Ⅱ and collagen Ⅺ and are referred to as collagen Ⅱ-containing fibrils. Likewise, the fibrils in fibrous tissues are comprised of collagen Ⅰ and collagen Ⅴ and are referred to as collagen Ⅰ-containing fibrils. Both collagen Ⅰ and collagen Ⅱ require their respective collagen partners for fibrillogenesis in vivo. Therefore, the absence of collagen Ⅺ results in major loss of collagen fibrils in cartilage [148]. Likewise, absence of collagen Ⅴ results in major fibrillogenesis defects in fibrous tissues [149]. Type Ⅲ collagen (another major fibrillar collagen) can occur in cartilaginous and fibrous tissues. Furthermore, collagen fibrils can be decorated with FACITs, which modify the interactive capabilities of fibril surfaces. Collagen Ⅴ is a minor fibrillar collagen which based on analyses of embryos lacking collagen Ⅴ plays a major role in the formation of fibrillar collagen matrices [149]. Lack of collagen Ⅴ leads to a mouse embryo without any fibrillar collagen formation, and the *Col5a1*^−/−^ mouse thus represents a severe embryonic fibrillar collagen phenotype. This phenotype has resulted in the view of collagen Ⅴ as a central nucleator of fibrillar collagens [150,151]. Despite the remarkable finding in *Col5a1*^−/−^ mice little progress has been made in understanding the molecular details underlying the phenotype. The role of collagen Ⅴ and the related collagen Ⅺ in collagen fibrillogenesis in adult tissues in situations of wound healing and tissue- and tumor fibrosis are also largely unexplored territories. As already mentioned, conditional deletion of FN in the liver did not interfere with fibrillar collagen matrix assembly [136]. When the hepatic stellate cells were studied in vitro, it was determined that collagen Ⅴ was needed for the fibrillar assembly of collagen I, supporting the evidence that collagen Ⅴ takes part in a collagen fibrillogenesis in a nucleating event. In connection with collagen Ⅴ’s role as a nucleator the obvious question is the nature of the link that connects collagen Ⅴ to the cell surface. Likely candidates for this role include, members of the integrin family, which directly via collagen-binding integrins or indirectly via COLINBRI-mediated mechanisms connect to non-collagen-binding integrins. So far, inactivation of integrins has not resulted in a phenotype corresponding to the collagen Ⅴ-deficient mice, and collagen-binding integrin deficient mice, in general, have mild phenotypes with no observed embryonic lethality [19]. Another interesting aspect relates to what region of collagen Ⅴ interact with cell surfaces during embryogenesis. If one assumes that heterotypic fibril formation occurs at the cell surface and based on existing models where collagen Ⅴ triple helix forms heterotypic alloy fibrils with collagen Ⅴ heterotrimers hidden in the interior of the fibril, with just the amino terminal col2 domain protruding, it is tempting to speculate that the interaction with cell surfaces occurs via col2 domain [28,149,152] (Figure 2). This is an area open for future studies.

## 4. Who Is Leading the Way—Fibronectin or Collagen—Or a Joint Venture?

The relative contribution of the FN-mediated assembly is predicted to vary with the total levels of collagen receptors present on cells. In cells lacking collagen receptors altogether, like the embryonic *Fn^−/−^* cells, the contribution of α5β1 integrin is significant when exogenous FN is provided [110]. In cells with both FN and collagen receptor present, both types of receptors seem to contribute to collagen fibrillogenesis [109]. As the matrix stiffens, as often is the case in tissue and tumor fibrosis, the levels of individual integrin receptors will be subjected to stiffness-dependent regulation of expression, most likely also affecting the relative contribution of the different integrins to collagen fibrillogenesis. Cells engineered to lack the classical α5β1 integrin, like recent α5 knock out pancreatic ductal adenocarcinoma CAFs [125] offers another extreme with minimal FN fibrillogenesis, but where collagen-binding integrins are predicted to play a major role in cell surface-assisted collagen fibrillogenesis. The FN and collagen matrix assembly processes are thus closely linked. In studies where FN assembly seems to depend on collagen fibrillogenesis, we predict that the levels of functional FN receptors are low. Once again, for a fuller mechanistic understanding, it thus seems central to establish the total repertoire of collagen and FN integrin receptors.

In future studies it will be useful to further study fibrillar adhesions and collagen-binding integrin-mediated collagen fibrillogenesis.

In summary:

1. Collagens are assembled and secreted as procollagen molecules, and pro-peptides can be cleaved off extracellularly in close association with cells surface. FN can aid in BMP-1-mediated extracellular collagen Ⅰ pro-peptide cleavage and in this way help to facilitate collagen I fibrillogenesis at the cell surface.
2. Binding to cell surface integrins enable cell-directed collagen fibrillogenesis either directly, by collagen-binding integrins or indirectly, by being captured by FN fibrils which have first been assembled by FN receptors.
3. The relative contribution of FN and collagen-binding integrins to collagen fibrillogenesis most likely vary in a dynamic manner during physiological and pathological processes.
4. Collagens Ⅴ/Ⅺ have a nucleating role in formation of heterotypic fibrils and might be the first collagens to bind to cell surface via collagen-binding integrins.

The final properties of a tissue-specific interstitial collagen matrix after initial fibrillogenesis has occurred are determined by ratios of different collagens in the heterotypic alloy fibrils in addition to the properties being influenced by interactions with other molecules including tenascin-Ⅹ, periostin, decorin, matrilins and cartilage oligomeric matrix protein [85,153,154,155]. With new technology such as CRISPR/Cas9 we will be able to stably knock out genes at an increased rate and look at their effects on matrix assembly in 2D and 3D environments of different stiffness. With improved resolution of microscopic techniques, we will also be able to obtain more detailed information. Use of super-resolution microscopy has recently shown that the FN fibrils are not continuous but consist of nanodomains [156]. As we learn more, it is likely that the unexpected will continue to surprise us. We are hopeful that in the not-too-distant future, we will have a clearer picture of how collagen fibrils form at the cell surface.
